# Topical Coenzyme Q10 demonstrates mitochondrial-mediated neuroprotection in a rodent model of ocular hypertension

**DOI:** 10.1016/j.mito.2017.05.010

**Published:** 2017-09

**Authors:** Benjamin Michael Davis, Kailin Tian, Milena Pahlitzsch, Jonathan Brenton, Nivedita Ravindran, Gibran Butt, Giulia Malaguarnera, Eduardo M. Normando, Li Guo, M. Francesca Cordeiro

**Affiliations:** a Department of Visual Neuroscience, UCL Institute of Ophthalmology, London EC1V 9EL, United Kingdom; b Western Eye Hospital, Imperial College London, United Kingdom

**Keywords:** Glaucoma, Retinal ganglion cell, Apoptosis, Neuroprotection, P-glycoprotein, Membrane biophysics

## Abstract

Coenzyme Q10 (CoQ10) is a mitochondrial-targeted antioxidant with known neuroprotective activity. Its ocular effects when co-solubilised with α–tocopherol polyethylene glycol succinate (TPGS) were evaluated. *In vitro* studies confirmed that CoQ10 was significantly protective in different retinal ganglion cell (RGC) models. *In vivo* studies in Adult Dark Agouti (DA) rats with unilateral surgically-induced ocular hypertension (OHT) treated with either CoQ10/TPGS micelles or TPGS vehicle twice daily for three weeks were performed, following which retinal cell health was assessed *in vivo* using DARC (Detection of Apoptotic Retinal Cells) and post-mortem with Brn3a histological assessment on whole retinal mounts. CoQ10/TPGS showed a significant neuroprotective effect compared to control with DARC (*p* < 0.05) and Brn3 (*p* < 0.01). Topical CoQ10 appears an effective therapy preventing RGC apoptosis and loss in glaucoma-related models.

## 1 Introduction

Glaucoma is a progressive neurodegenerative eye disorder estimated to affect 60 million people worldwide (Cook and Foster, 2012, Tham et al., 2014). Glaucoma involves the progressive loss of retinal ganglion cells (RGCs) and their axons, which results in visual field abnormalities and ultimately blindness if left untreated (Garcia-Valenzuela et al., 1995, Quigley et al., 1995). Elevated intraocular pressure (IOP) is presently the only modifiable disease risk factor (Weinreb and Khaw, 2004, Lee et al., 2014a). However, recognition of a subset of glaucoma patients who continue to exhibit visual decline despite therapeutically well-controlled IOP has led to the realisation that novel therapeutic paradigms for this condition are urgently required (Resnikoff et al., 2004).

RGC loss in glaucoma is predominantly thought to occur *via* elevated apoptosis (a type of programmed cell death) (Quigley et al., 1995, Cordeiro et al., 2010) which is mainly mitochondrial dysfunction mediated (Lee et al., 2014a, Ju et al., 2008, Park et al., 2011). While the primary site of injury is thought to occur at the site of the RGC axon in the optic nerve, (Quigley et al., 1977, Minckler et al., 1977, Quigley et al., 1981, Knox et al., 2007) the resulting loss of RGCs (primary degeneration) can also lead to the secretion of pro-apoptotic factors resulting in secondary neurodegeneration and the death of neighbouring RGCs (Davis et al., 2016a). Although the exact mechanism of glaucoma progression remains to be elucidated, elevated oxidative stress has been suggested to contribute to glaucoma pathogenesis (Tezel et al., 2005, Yuki et al., 2010). Mitochondria are a source and target of oxidative stress and therefore are key in the development of neuroprotective strategies for RGC preservation in glaucoma (Chrysostomou et al., 2013).

Coenzyme Q10 (CoQ10) is a mitochondrial targeted antioxidant that plays an essential role in the normal function of the electron transport chain. CoQ10 has been reported to exhibit neuroprotective activity in a range of disorders including; cerebral ischemia, (Ahmed et al., 2015) Parkinson's disease and Huntington's disease (Klongpanichapak et al., 2006). In addition to its role as an antioxidant, CoQ10 is also reported to protect against glutamate excitotoxicity *in vivo* through the inhibition of mitochondrial depolarization (Papucci et al., 2003, Lee et al., 2014b).

Concentrations of CoQ10 in the human retina are reported to decline by up to 40% with age (Qu et al., 2009). The poor aqueous solubility (Fato et al., 2010) and low bioavailability of CoQ10, due in part to its interactions with the multi-drug efflux pump P-glycoprotein (P-gp), have limited the development of topically active formulations of this drug (Hirano and Iseki, 2008). The interaction of CoQ10 with P-gp, expressed in both corneal epithelial cells (Vellonen et al., 2010) and RGCs (Duncan et al., n.d.) suggests that co-administration of CoQ10 with a P-gp inhibitor would likely enhance the topical delivery and pharmacological effects of this drug (Hirano and Iseki, 2008). α-Tocopherol is a form of vitamin E best known for its role as a lipid soluble antioxidant but is well-documented to inhibit P-glycoprotein (P-gp) activity (Wu et al., 2007, Davis et al., 2015). The mechanism of α-Tocopherol mediated P-gp inhibition is poorly understood but has recently been suggested to occur as a result of indirect modulation of the membrane dipole potential (Davis et al., 2015).

Formulation of CoQ10 into micelles using the vitamin E derivative D-α-Tocopherol polyethylene glycol 1000 succinate (TPGS) has previously been reported to deliver micromolar concentrations of CoQ10 to the vitreous in patients 1 h after administration (Fato et al., 2010). The present study sought to investigate the mechanism of α-Tocopherol mediated P-gp inhibition and assess the neuroprotective effects of CoQ10 and TPGS using immortalised and primary mixed retinal cultures (Galvao et al., 2014, McCarthy et al., 2004). Finally, the efficacy of topically applied CoQ10/TPGS micelles was next evaluated *in vivo* using the well-established Morrison's ocular hypertension model (OHT) (Morrison et al., 1997) and *in vivo* DARC (Cordeiro et al., 2017) and Brn3a-RGC immunohistochemistry as endpoints (Galvao et al., 2013, Davis et al., 2016b).

## 2 Methods

### 2.1 Cell culture

Both primary murine retinal mixed cultures (pMC) and an immortalised retinal neuronal (RN) cell line (RGC5, a gift from Dr. Neeraj Agarwal, Department of Cell Biology and Genetics, UNT Health Science Centre, Fort Worth, TX) were used. These cells express retinal neuronal proteins Thy-1, Brn3a, and β3 tubulin (Krishnamoorthy et al., 2001, Burugula et al., 2011, Nadal-Nicolás et al., 2009), and are known to be similar to the 661w photoreceptor cell line and RGCs (Al-Ubaidi, 2014, Van Bergen et al., 2009, Krishnamoorthy et al., 2013). RN were cultured in Dulbecco's modified Eagle's medium (DMEM; Invitrogen, Paisley, UK), supplemented with 10% heat-inactivated fetal bovine serum (Invitrogen), 100 U/mL penicillin and 100 mg/mL streptomycin. Primary murine (C57BL/6) mixed retinal cultures were isolated from P1 pups and neuronal cells isolated by incubation in a solution containing 10 units of papain/mL, and cultured in DMEM supplemented with 5% fetal bovine serum (Invitrogen, UK), 100 U/mL penicillin, 100 μg/mL of streptomycin and 0.292 mg/mL glutamine (Gibco, UK), 7.5% sterile dH20 and 1.5 mM KCl (Sigma-Aldrich, UK). The medium was changed completely on day 1 and 50% refreshed on day 2. Cells were used for experiments on day 3.

### 2.2 P-glycoprotein activity assessment

Analysis of P-gp activity was performed as previously described (Ohashi et al., 2006). Briefly, RN cells were seeded at 4000 cells/well in a 96 well plate for 24 h. On the day of the study, cell monolayers were washed before treatment with varying concentrations of TPGS or verapamil hydrochloride (Sigma-Aldrich), a known P-gp inhibitor for 10 min and incubated for 10 min at 37 °C. After this time, cells were incubated with the P-gp substrate calcein-AM (Invitrogen) for 60 min before P-gp activity was measured by quantifying calcein fluorescence using excitation and emission wavelengths of 485 nm and 530 nm respectively (Safire plate reader). Percentage P-gp activity at each concentration of drug was determined using Eq. (1);

(1) $$\mathrm{Pgp}\;\text{activity}\;\left(\%\right)=100-\frac{\left({\mathrm{RFU}}_{\text{test}}-{\mathrm{RFU}}_{\mathrm{BK}}\right)}{\left({\mathrm{RFU}}_{\mathrm{MAX}}-{\mathrm{RFU}}_{\mathrm{BK}}\right)}$$

where; RFU_test_ is the fluorescence in the presence of test compound, RFU_BK_ is the fluorescence in the absence of test compounds and RFU_MAX_ is the fluorescence in the presence of 66 μM verapamil which induced maximal P-gp inhibition. EC_50_ values were determined by fitting results to four-parameter dose response curves.

### 2.3 Dipole potential assessment

RN cultures were seeded at 4000 cells/well in a 96 well plate and permitted to settle for 24 h before washing well before labelling with 0.5 μM of the fluorescent probe di-8-ANEPPs (Invitrogen, from 2 mM stock solution in ethanol) for 1.5 h in phenol-red free DMEM (Sigma-Aldrich) (Davis et al., 2015). After this time the ratiometric di-8-ANEPS fluorescence intensity at excitation of 420/520 nm and emission of 670 nm using a Safire plate reader for each cell population was recorded before and 10 min after cells were treated with varying concentrations of TPGS for 10 min. The change in fluorescence ratio of di-8-ANEPPS indicates a change in the membrane dipole potential on addition of an agent of interest. The dissociation constant (K_d_) of the interaction of TPGS for neuronal cells was determined by fitting the change in di-8-ANEPPs fluorescence ratio to a hyperbolic binding equation as described previously (Davis et al., 2010).

### 2.4 Immunocytochemistry

pMC were fixed in 4% paraformaldehyde for 15 min before washing twice with PBS and permeabilizing in PBS plus 0.1% Tween-20. Cells were blocked with PBS containing 3% bovine serum albumin (BSA, Sigma-Aldrich, UK) for 1 h prior to incubation with primary antibodies overnight at 4 °C (diluted in PBS containing 3% BSA; see Table 1 for details of antibodies used), followed by the appropriate Alexa Fluor 488 nm or 555 nm secondary antibody for a further hour at a 1:1000 dilution (Life technology, UK). Cells were subsequently washed twice with PBS, before addition of 5 μg/mL cell permeable dye Hoechst 33342 (Molecular Probes, Eugene, OR, USA) for 5 min at room temperature prior to visualisation. Then mounted with mowiol (Merck, UK) and were observed under a confocal fluorescence microscope (LSM 700, Carl Zeiss MicroImaging GmbH, Jena, Germany).

**Table 1:** Antibodies source and optimized dilutions.

Antibody	Company	Cat.	Host species	Dilution
Brn3a	Abcam	AB81213	Rabbit	1:200
Thy-1	Abcam	AB225	Mouse	1:500
RBPMS	Abcam	AB152101	Rabbit	1:500
γ-synuclein	Abcam	AB55424	Rabbit	1:1200

### 2.5 Reverse transcription PCR assay

To test pMC for retinal neuronal marker expression, total RNA was extracted from primary mixed retinal cultures using RNeasy mini kit following manufacturer's specifications (Qiagen, UK). Complementary DNA (cDNA) synthesis was conducted by QuantiTect Reverse Transcription (Qiagen) according to manufacturer's protocol. The PCR reaction was conducted using the GoTaq G2 DNA polymerase kit (Promega, UK). Primers and cycle conditions are summarised Table 2.

**Table 2:** Summary of PCR primers.

Gene	NCBI ref. (Murine mRNA[cDNA])	Primers Forward (5′- > 3′) Reverse(3′- > 5′)	PCR product length	Tm (°C)
Thy-1	NM_009482.3	TGAGGGAAGTTGGACTGTGC CCCTTCCTGCACGGACTTAG	405	60
Brn3a (Pou4f1)	NM_011143.4	CCTCGTCTGAGAAGATCGCC AACAACGCCTACCCAGAGTG	790	60
γ-synuclein	NM_011430.3	CACACTGAATGCCCTGCCTA ACAGCAGCATCTGATTGGTGA	156	60

### 2.6 Oxidative cytotoxicity evaluation and cell viability assays

pMC were plated at 30,000 cell/well in 96-well plates for 24 h. After this time cells were treated with either 20 μM CoQ10 with 57 μM TPGS, or 57 μM TPGS only (vehicle control) for 2 h. The molar ratio of CoQ10 and TPGS chosen was the same as that present in the micelle formulation subsequently used *in vivo*. After this time, treatments were removed before application of varying concentrations of cytotoxic insults (DMSO or paraquat, Sigma-Aldrich, UK) which were incubated for 24 h (5% CO_2_, 37 °C). Cell viability was then assessed using the Alamarblue (Invitrogen, UK) assay according to manufacturer's instructions. Briefly, the Alamarblue solution was added to each well to a final concentration of 10% v/v. Cells were incubated for 4 h at 37 °C before fluorescence was recorded using a Safire plate reader (excitation of 530 nm and emission of 590 nm) and cell viability determined as previously described (Lancaster and Fields, 1996). Results presented are averages of at least three independent experiments.

### 2.7 Animals

All animal experiments were performed with procedures approved by the U.K. Home Office and in compliance with the ARVO Statement for the Use of Animals in Ophthalmic and Vision Research. For *in vivo* assessment of experiments: in total 20 Adult male Dark Agouti (DA) rats (Harlan Laboratories, UK) weighing 150 to 200 g were housed in an air-conditioned, 21 °C environment with a 12 h light-dark cycle (140–260 lx), where food and water were available *ad libitum*.

### 2.8 Ocular hypertension model

Ocular hypertension was surgically induced in the left eye of 20 DA rats as described previously (Morrison et al., 1997). Procedures were conducted under general anaesthesia using a mixture of 37.5% Ketamine (Pfizer Animal Heath, Exton, PA), 25% Dormitol (Pfizer Animal Heath, Exton, PA) and 37.5% sterile water, at 2 mL/kg administered intraperitoneally. Briefly, 50 μL of hypertonic saline solution (1.8 M) was injected into the two episcleral veins using a syringe pump (50 μL/min; UMP2; World Precision Instruments, Sarasota, FL, USA). A propylene ring with a 1 mm gap cut from the circumference was placed around the equator to prevent injected saline outflow from other aqueous veins. The IOP from both eyes of each rat was measured at regular intervals using a TonoLab tonometer (Tiolat Oy, Helsinki, Finland) under inhalational anaesthesia (0.4% isoflurane in oxygen). Daily administration of topical CoQ10/TPGS micelles (0.5% w/v TPGS with 0.1% CoQ10 w/v in PBS, pH 7.4) or TPGS only micelles (0.5% w/v TPGS, vehicle control) was performed in DA rats (two 30 μL drops/day 5 min apart at 10 am each day) starting two days prior to model induction and continuing until model termination (21 days post IOP elevation). Animals underwent DARC imaging before sacrifice three weeks after unilateral IOP elevation.

### 2.9 Detection of apoptotic retinal cells

Fluorescently labelled Annexin A5 (Anx776, (Cordeiro et al., 2017)) was given by intravitreal administration as described previously (5 μL of 0.4 μg/mL) (Cordeiro et al., 2010, Galvao et al., 2013, Guo et al., 2014). *In vivo* DARC imaging was performed using a modified cSLO (Heidelberg Retina Angiograph 2, Heidelberg Engineering, Dossenheim, Germany) (Cordeiro et al., 2004, Maass et al., 2007) and a 55° field of view centred on the optic disc (Cordeiro et al., 2004, Maass et al., 2007). No complications or intraocular side effects associated with topical treatments were recorded.

### 2.10 Brn3a immunohistochemistry and confocal microscopy

Brn3a labelling of RGCs in retinal whole mounts was completed as described previously (Davis et al., 2016a). Briefly, eyes were enucleated upon sacrifice and fixed in 4% paraformaldehyde at 4 °C overnight before dissecting retinal whole mounts. Whole mounts were stained for the RGC specific nuclear-localised transcription factor Brn3a using an anti-mouse mAb (1:500, Merck Millipore, Darmstadt, Germany) and examined under confocal microscopy (LSM 710, Carl Zeiss MicroImaging GmbH, Jena, Germany). Each retinal whole mount was imaged as a tiled z-stack at × 10 magnification which was used to generate a single plane maximum projection of the RGC layer in each retina for subsequent analysis. Each whole mount image was manually orientated so that the superior retina was towards the top of the image using *in vivo* cSLO imaging of retinal vasculature as a reference. Retinal image acquisition settings were kept constant for all retinas imaged, allowing comparison of Brn3a expression in each experimental group as previously described (Nadal-Nicolás et al., 2012). Automated quantification of Brn3a labelled RGCs in retinal whole mounts was completed as described previously (Davis et al., 2016a). Naïve Brn3a whole retinal counts from DA rats (Fig. 6) was obtained from our previous work (Davis et al., 2016a).

### 2.11 Statistical analysis

All data were analysed with the Student's *t*-test or ANOVA with posthoc testing using GraphPad Prism 5 (GraphPad Software, Inc., La Jolla, CA, USA) as appropriate. Data were presented as means ± SE and *p* < 0.05 was considered significant.

## 3 Results

### 3.1 The vitamin E derivative TPGS modulates P-glycoprotein activity and membrane dipole potential over the same concentration range in immortalised neuronal cells

**Fig. 1:**
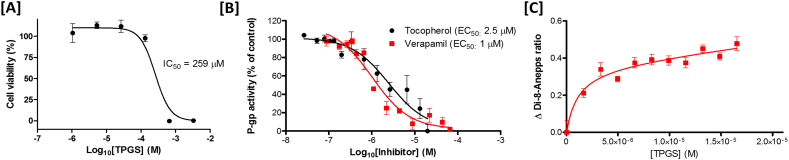
The P-gp inhibition activity of TPGS in a neuronal cell line closely matches its dipole potential modulating effects [A] Dose response curve (AlamarBlue) for a retinal neuronal cell line after 18 h incubation with TPGS (n = 3). [B] Comparison of the effect of TPGS and verapamil hydrochloride on P-gp activity in the same retinal neuronal cell line. Data expressed as the mean ± SE (n = 6). The figure shows a dose-dependent decrease in P-gp activity with both verapamil hydrochloride and TPGS fit four parameter dose-response curves. [C] Change in membrane dipole potential on titration of TPGS into retinal neuronal cell line as determined by di-8-ANEPPs fit best to a hyperbolic binding equation with a dissociation constant similar to the IC_50_ of TPGS for P-gp (2.48 ± 0.06 μM *versus* 2.22 ± 0.03 μM respectively). Results are means ± SE.

The effect of TPGS on the viability of immortalised RN cells was first established using the AlamarBlue viability assay (Fig. 1A). The IC_50_ of TPGS after 24 h incubation was found to be 259 ± 14 μM with no significant reduction in cell viability observed up to TPGS concentrations of 132 μM. The calcein-AM P-gp activity assay (Fig. 1B) determined the IC_50_ of verapamil as 1.03 ± 0.02 μM which is similar to that reported elsewhere in the literature (Kishimoto et al., 2016). The IC_50_ of TPGS was found to be 2.48 ± 0.06 μM, in agreement with reports in the existing literature that this molecule is a P-gp inhibitor despite not being a direct P-gp substrate (Collnot et al., 2010). Using the same model, the influence of TPGS on the membrane dipole potential was investigated (Fig. 1C). The interaction of TPGS with this neuronal cell line was found to induce a marked decline in the membrane dipole potential in a similar manner to that previously reported for α-tocopherol which fit a hyperbolic binding equation with a dissociation constant of 2.22 ± 0.03 μM. The striking similarity between the IC_50_ of TPGS for P-gp and the effect of TPGS on the membrane dipole potential provide further evidence to support the hypothesis that modulation of membrane dipole potential indirectly modulates P-gp activity.

### 3.2 Coenzyme Q10 micelles are neuroprotective in vitro against established models of mitochondrial-mediated neurotoxicity in rodent primary mixed retinal cultures

**Fig. 2:**
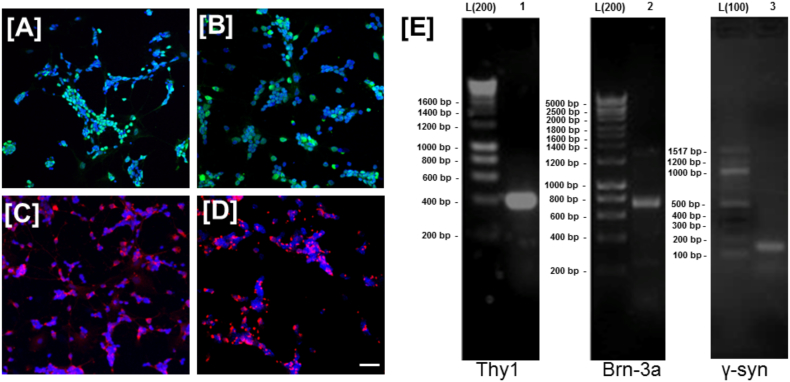
Characterization of primary mixed murine retinal cultures enriched in RGCs. Immunostaining of primary murine cultures reveals a high concentration of cells labelled with RGC specific markers [A] Brn3a, [B] γ-synuclein, [C] RBPMS [D] Thy-1. Hoechst nuclear staining (blue) with immunostaining (FITC/TRITC). Scale bar = 20 μm, × 10 magnification. [E] Results were confirmed with reverse-transcriptase PCR using primers against (1) Thy-1, (2) Brn3a and (3) γ-synuclein. Band sizes were confirmed by comparison to appropriate molecular weight ladder; 200 bp (L200) or 100 bp (L100). No bands were detected in primary cell-free controls (data not shown) ruling out primer-dimers. (For interpretation of the references to color in this figure legend, the reader is referred to the web version of this article.)

Primary mixed murine retinal cultures were firstly characterised immunohistochemically (Fig. 2A–D) and by mRNA expression using PCR (Fig. 2E). A proportion of mixed retinal cultures were found to label with the RGC specific markers Brn3a, γ-synuclein, RBPMS and Thy-1 and expression of RGC specific markers was confirmed by PCR (Fig. 2E).

**Fig. 3:**
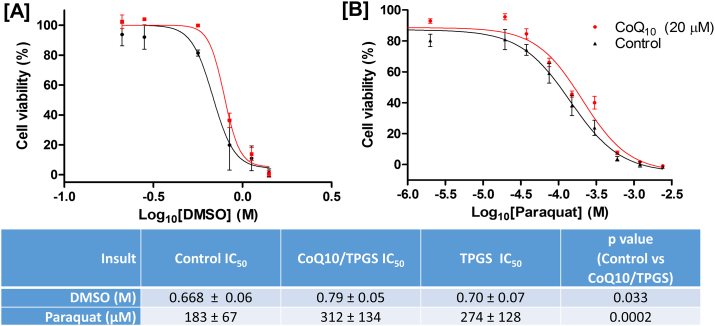
CoQ10/TPGS micelles but not TPGS alone are neuroprotective against mitochondrial targeted cytotoxic insults in mixed murine retinal cultures containing RGCs. Pre-treatment of primary mixed murine retinal cell cultures with CoQ10/TPGS micelles (20 μM CoQ10 and 57 μM TPGS) but not equivalent concentrations of TPGS only significantly (two-way ANOVA, *p* = 0.033 and *p* = 0.0002 respectively) reduced the susceptibility of these cells to [A] DMSO and [B] paraquat-induced cytotoxicity.

Pre-treatment of pMC cultures with CoQ10/TPGS micelles was found to significantly reduce cell death induced by DMSO and paraquat (unmatched two-way ANOVAs, *p* = 0.031 & *p* = 0.002 respectively) (Fig. 3A–B). Treatment of cells with equivalent concentrations of TPGS alone did not elicit a significant neuroprotective effect in either cytotoxic model.

### 3.3 Topically applied Coenzyme Q10 micelles reduce RGC apoptosis in the Morrison's model of ocular hypertension independent of IOP

**Fig. 4:**
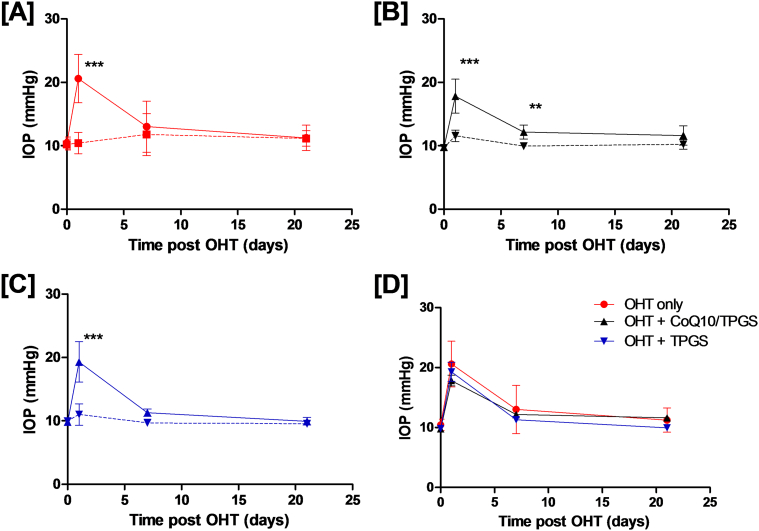
CoQ10/TPGS or TPGS eye drop administration did not significantly affect IOP elevation induced by the OHT model. IOP profiles in DA rats after induction of OHT demonstrate a significant increase in IOP *versus* contralateral eyes [A–C] (Two-way repeated measures ANOVA with Bonferroni post-test, ***p < 0.001, **p < 0.01). Treatment of eyes with topical administration of CoQ10/TPGS [B] or TPGS only micelles [C] did not significantly alter the IOP profiles compared on OHT induction (two-way repeated measures ANOVA with Bonferroni post-test *versus* OHT model, *p* > 0.05) [D] suggesting any neuroprotective activity of treatments was a result of IOP independent effects. Results are mean ± SD.

Having established the neuroprotective potential of CoQ10/TPGS micelles *in vitro*, we next sought to determine whether topical application of CoQ10/TPGS micelles could induce neuroprotection using a well-established rodent model of experimental glaucoma. Induction of OHT in DA rats resulted in an increase in IOP (Table 3), which peaked 1-day post-surgery in all treatment groups (Fig. 4A) and returned to baseline levels by the three-week time point. No significant change in IOP was observed in contralateral eyes (Fig. 4A–C), in agreement with previous studies (Davis et al., 2016a). Topical instillation of CoQ10/TPGS or TPGS only micelles did not cause a significant change in IOP profile compared to untreated OHT, suggesting any other effects observed were independent of IOP (Fig. 4D).

**Table 3:** Mean IOP measurements and integral IOP (± SD) for each treatment group in this study.

Time post OHT induction (days)	OHT only	OHT (co-eye)	OHT + CoQ10/TPGS	OHT + CoQ10/TPGS (co-eye)	OHT + TPGS	OHT + TPGS (co-eye)
0	10.4 (1.0)	10.2 (0.88)	9.8 (0.2)	9.7 (0.2)	9.9 (0.3)	10.1 (0.4)
1	20.6 (3.8)	10.4 (1.7)	17.8 (2.7)	11.6 (0.9)	19.0 (2.8)	11.1 (1.5)
7	13 (4.0)	11.8 (3.3)	12.1 (1.1)	9.9 (0.4)	11.3 (0.6)	9.7 (0.1)
21	11.2 (2.0)	11.1 (1.2)	11.6 (1.5)	10.2 (0.8)	10.0 (0.6)	9.5 (0.3)
Integral IOP (mmHg/day)	286.0 (42.4)	237.3 (43.0)	270.2 (27.3)	216.2 (7.7)	255.1 (14.5)	206.9 (5.4)

**Fig. 5:**
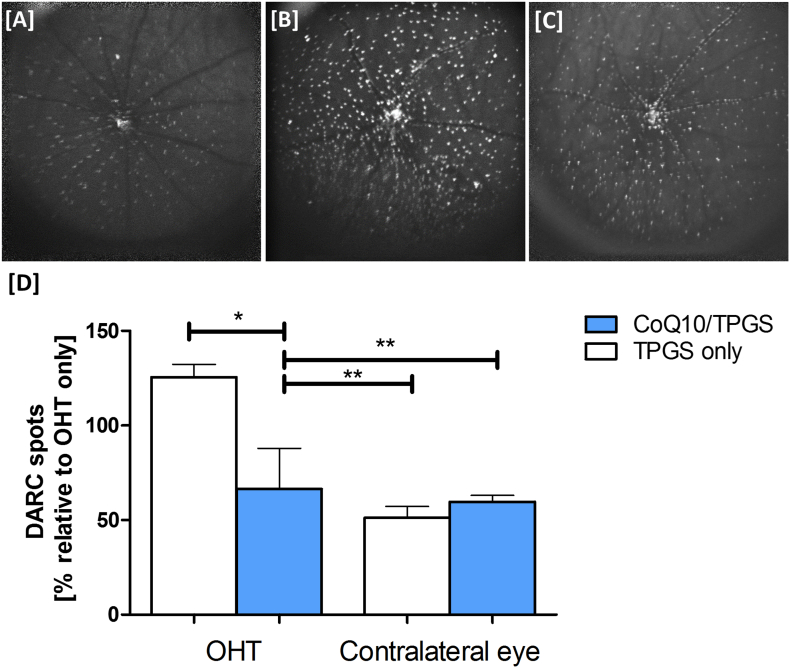
*In vivo* detection of apoptotic retinal cells using DARC reveals CoQ10/TPGS micelles are significantly neuroprotective in the Morrison's OHT model. [A] Sample DARC image from CoQ10/TPGS treated DA rats exhibiting fewer apoptotic retinal cells (bright spots) than [B] eyes receiving TPGS only micelles or [C] OHT only eyes. [D] CoQ10/TPGS treatment was found to significantly reduce the DARC spot count when quantified by masked observers (one-way ANOVA with Tukey posthoc test, *p* < 0.05). Results are means ± SE.

Three weeks after surgical induction of OHT, animals had DARC imaging performed. The number of apoptotic RGCs was quantified from acquired retinal images by recording mean counts from two trained masked observers. A significantly lower number of apoptotic retinal cells was detected in OHT eyes treated with CoQ10/TPGS micelles compared to those treated with micelles containing only TPGS (one-way ANOVA with Tukey posthoc test, *p* < 0.05, Fig. 5). The number of apoptotic cells detected after treatment with CoQ10/TPGS micelles was similar to that detected in contralateral unoperated eyes.

**Fig. 6:**
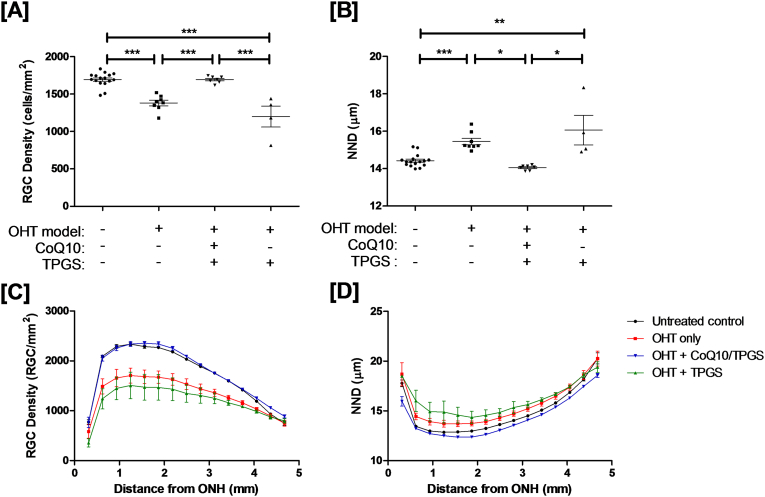
Retinal ganglion cell survival after Morrison's OHT model in the DA rat is significantly enhanced by topical treatment with CoQ10/TPGS micelles. [A] OHT model induction led to a significant decline in RGC density (and increase in nearest neighbour distance) which was not recovered by treatment with TPGS micelles alone. Daily topical administration of CoQ10/TPGS micelles resulted in a significant preservation in RGC populations (one-way ANOVA with Tukey posthoc tests, *p* < 0.001). On dividing the retina into a series of 15 concentric non-overlapping rings (as described in (Davis et al., 2016a)), most pronounced RGC preservation occurs in the central retina [C, D].

RGC loss was evaluated by whole-retinal flat mounts labelled with Brn3a. CoQ10/TPGS but not TPGS treatment alone could protect rat retinal RGCs against IOP-induced apoptosis as indicated by the preservation in RGC density (Fig. 6A & B) and nearest neighbour distance (Fig. 6C & D) in the CoQ10/TPGS treated groups *versus* TPGS only or untreated (OHT only) controls.

## 4 Discussion

This study uses both *in vitro* and *in vivo* mitochondrial-mediated neurotoxicity models to successfully demonstrate the neuroprotective activity of CoQ10/TPGS compared to TPGS alone. Furthermore, twice-daily topical instillation of CoQ10/TPGS micelles was found to be significantly neuroprotective against RGC loss in a well-established rat model of OHT using *in vivo* and *ex-vivo* endpoints.

The findings also suggest that the antioxidant activity of TPGS alone was insufficient to protect an immortalised neuronal cell line from insults generating mitochondrial oxidative stress, such as DMSO and paraquat. This is in agreement with previous work which reported that co-administration of CoQ10 with the α-tocopherol derivative trolox enhances the neuroprotective activity of CoQ10 *in vitro* (Nakajima et al., 2008). The authors postulated the beneficial effect of vitamin E/CoQ10 co-therapy is a result of both agents having a synergistic antioxidant potential (Constantinescu et al., 1994). The reactivity of CoQ10 towards peroxyl radicals is reported to be much lower than that of α-tocopherol (0.33 × 10^4^ M^− 1^·s^− 1^ *versus* 3.3 × 10^6^ M^− 1^·s^− 1^ respectively (Sohal, 2004)). However, the ability of CoQ10 to regenerate reduced α-tocopherol in the mitochondrial membrane (Sohal, 2004) and previous observations that supplementation with CoQ10 increases mitochondrial α-tocopherol concentration but not *vice-versa* (Lass and Sohal, 2000) could explain this effect. TPGS is not an antioxidant, first requiring decomposition by cellular esterases to liberate α-tocopherol, perhaps reducing the effective concentration of this antioxidant (Carini et al., 1990).

CoQ10 has been used in several treatment trials of retinal disorders. A randomized, double-blind, placebo-controlled clinical trial of 106 AMD (age-related macular degeneration) patients, reported improvements in visual function and retinal lesions after 12 months of oral CoQ10 therapy (20 mg/day) combined with mitochondrial targeting therapies Acetyl-l-Carnitine (200 mg/day) and n-3 Fatty acids (20 mg/day) (Feher et al., n.d.). More recently, the effects of topical CoQ10 therapy (2 drops/day) in combination with vitamin E TPGS (CoQun) was assessed in 22 open-angle glaucoma patients receiving β-blocker *versus* 21 patients receiving β-blocker monotherapy alone. This study reported a beneficial effect of CoQun therapy on electrophysiological functional tests including pattern electroretinography and visual cortical responses after 12 months Coqun therapy *versus* controls (Parisi et al., 2014).

The mechanism by which CoQ10 is thought to elicit neuroprotection is suggested to be a result of a combination of its well-documented antioxidant activity (Turunen et al., 2004), mechanical stabilisation of membrane structure reducing the risk of mitochondrial depolarisation (Sévin and Sauer, 2014) or *via* its Ca^2 +^ buffering activity (Bogeski et al., 2011), important as an increase in intracellular Ca^2 +^ is associated with apoptosis induction (Pinton et al., 2008). The ability of CoQ10 to inhibit glutamate excitotoxicity has been attributed to the reduction in expression of NR1 and NR2A subunits of *N*-methyl-d-aspartate receptor in the DBA/2J murine glaucoma model (Lee et al., 2014b). As both oxidative stress and glutamate excitotoxicity have been suggested to contribute to glaucoma pathogenesis, CoQ10 presents an intriguing glaucoma therapy (Davis et al., 2016c). Particularly as CoQ10 levels in the retina decline by approximately 40% with age, which may be associated with the onset of retinal disease (Qu et al., 2009).

*In vitro*, CoQ10 treatment was found to have a more pronounced effect on DMSO than paraquat IC_50_ values. A possible explanation for the observation is that while both DMSO and paraquat induce oxidative stress *via* affecting mitochondrial mediated respiration (Galvao et al., 2014, Yuan et al., 2014, Castello et al., 2007), we recently reported that DMSO can also induce an increase in cytoplasmic calcium resulting in BAX-mediated apoptosis induction. Coenzyme Q10 has recently been reported to bind and transport Ca^2 +^ across membranes (Bogeski et al., 2011). The authors postulate that in addition to its anti-oxidant properties, coenzyme Q10 could therefore act as a cytosolic Ca^2 +^ buffer so protecting mitochondria from elevated cytosolic Ca^2 +^ levels.

In support of a RGC neuroprotective mechanism, local ocular and systemic administration of CoQ10 (most commonly in conjunction with vitamin E derivatives) have been reported to offer retinal neuroprotective activity against models of retinal damage. Intravitreal and topical administration of CoQ10 has been reported to protect against retinal damage caused by IOP-induced ischemia or staurosporine by preventing glutamate-induced excitotoxicity and RCG apoptosis respectively (Nucci et al., 2007, Guo and Cordeiro, 2008). More recently, topical CoQ10 was found to elicit RGC neuroprotection over and above its antioxidant activity in a UV-induced rat model of retinal damage through inhibition of mitochondrial depolarization after topical instillation, (Papucci et al., 2003, Lulli et al., 2012) Nakajima et al. reported that systemic administration of CoQ10 (10 mg/kg) protected retinal cells against oxidative stress in an *in vivo* murine model of NMDA-induced retinal injury (Nakajima et al., 2008). Furthermore, in a transgenic DBA/2J murine glaucoma model, daily supplementation of the diet with 1% CoQ10 was found to promote RGC survival by 29% through decreasing Bax or increasing pBad protein expression and preserving mtDNA content and Tfam/OXPHOS complex IV protein expression in the glaucomatous retina (Lee et al., 2014b).

The protective effects of CoQ10 are not limited to neurons, with increasing reports that dietary supplement with CoQ10 therapy can also inhibit astroglial activation *via* mitochondrial-mediated effects, (Papucci et al., 2003, Lee et al., 2014b, Noh et al., 2013) which is increasingly recognised to play an important role in glaucoma pathology (Seitz et al., 2013). As a result, in addition to the aforementioned direct neuroprotective effects, CoQ10 may also elicit neuroprotective activity by acting on retinal glia. Administration of both DMSO (up to 5% v/v) and paraquat have previously been reported to promote astrocyte and glial toxicity *in vitro* (Yuan et al., 2014, Kim et al., 2008). Furthermore, subcutaneous administration of DMSO in P7 C57/BL/6By mice is reported to induce microglial activation in the brain (Saito et al., 2015) and administration of sub-toxic doses of paraquat in mice are reported to result in microglial activation prior to neurodegeneration (Purisai et al., 2007). In addition, a microglial inhibitory mechanism has recently been proposed in CoQ10 mediated protection against Aβ(1–42) induced cognitive dysfunction (Meneses et al., 2015) and pentylenetetrazol induced kindling epilepsy in mice (Bhardwaj and Kumar, 2016). In addition, with accumulating evidence for the involvement of amyloid beta in glaucoma pathology (Guo et al., 2007, Ito et al., 2012, Nizari et al., 2016) and growing recognition of mechanistic similarities between glaucoma and Alzheimer's disease (Gupta et al., 2016, Sivak, 2013), modulation of microglial activation by CoQ10 could contribute to the reported neuroprotective effects of this agent. Finally, reports of microglia activation in angiogenesis (Arnold and Betsholtz, 2013) and recent reports of microglial contribution to elevated basic fibroblast growth factor (bFGF) expression in the CNS after injury (Fujimaki et al., 2016) (perhaps *via* the ERK pathway (Lu et al., 2007, Ibrahim et al., 2011)), suggest a potential mechanism for the reported anti-angiogenic effects of CoQ10 (Choi et al., 2011, Jung et al., 2009, Sachdanandam, 2008) and its potential as a therapeutic for the treatment of age-related macular degeneration.

Beyond increasing the aqueous solubility and antioxidant potential of CoQ10, this study provides evidence to suggest that inhibition of P-gp activity may also play a role in the benefit of CoQ10/TPGS co-therapy. Inhibition of P-gp will reduce the efflux of extracellularly administered CoQ10, which is a recognised P-gp substrate (Hirano and Iseki, 2008). P-gp inhibition could, therefore, act to both increase the concentration of CoQ10 reaching intraocular tissues (*via* inhibition of P-gp in corneal epithelial cells, which contributes to the formidable corneal barrier to topically applied drugs (Dey et al., 2004)) and impede the removal of CoQ10 from neuronal cells in the retina.

Although the P-gp inhibiting activity of TPGS is well-established (Dintaman and Silverman, 1999, Constantinides et al., 2006), the mechanism of action is poorly understood. TPGS is known not to interact directly with P-gp (Collnot et al., 2010), suggesting an indirect mechanism of action. There has been a recent growth in interest in the indirect modulation of membrane protein function *via* non-specific (Type II) lipid-protein interactions (Richens et al., 2015). The membrane dipole potential describes an electrical potential which arises from the restricted orientation of dipoles within membrane lipids and water molecules of the membrane solvation shell and has a magnitude of ~ 300 mV (O'Shea, 2003). The ability of α-tocopherol to modulate the membrane dipole potential of cholesterol containing membrane microdomains has recently been suggested as a possible mechanism of indirect P-gp activity modulation (Davis et al., 2015). In the present study, titration of TPGS in an immortalised neuronal cell line was found to induce a dose-dependent change in the membrane dipole potential, which fitted to a hyperbolic binding equation with a dissociation constant strikingly similar to the concentration of TPGS required to inhibit 50% of P-gp activity in the same cell line. Together, this data provides further evidence to support dipole potential modulation as a mechanism for α-tocopherol mediated P-gp inhibition.

Topical instillation of CoQ10/TPGS micelles but not TPGS micelles alone was found to significantly reduce the number of apoptotic retinal ganglion cells three weeks after induction of the OHT model without affecting IOP, suggesting an IOP independent neuroprotective effect of topical CoQ10 therapy. These results were confirmed with Brn3a whole mount histology which indicated almost complete protection of RGCs in the OHT retina upon treatment with CoQ10/TPGS micelles *versus* TPGS only or untreated groups. The results of this study provide evidence to support the use of the DARC technique to provide a quantitative assessment of retinal apoptosis and monitor the efficacy of therapeutic interventions *versus* appropriate controls. The impressive neuroprotective effect of CoQ10/TPGS may be a result of treatment commencing two days before OHT induction, suggesting this therapy may be most effective for patients at risk of IOP spikes such as following posterior capsulotomy or in pigment dispersion and Posner-Schlossman syndromes.

## 5 Conclusion

In conclusion, this study presents evidence that topically instilled CoQ10/TPGS micelles can deliver neuroprotective concentrations of these antioxidants to the retina *in vivo* using an established rodent model of ocular hypertension. These findings are in agreement with recent literature which suggests that this formulation can be used to deliver therapeutically relevant concentrations of CoQ10 to the posterior ocular tissues in humans after topical instillation (Fato et al., 2010) and suggest the potential utility of this neuroprotective therapies for the treatment of glaucoma.

## Conflict of interest

MFC also holds patents pertaining the DARC technology. Visufarma holds patents regarding topical formulation of CoQ10.
