# Corrigendum to “Topical Coenzyme Q10 demonstrates mitochondrial-mediated neuroprotection in a rodent model of ocular hypertension” [Mitochondrion 36 (2017) 114–123]

**DOI:** 10.1016/j.mito.2019.05.005

**Published:** 2019-07

**Authors:** B.M. Davis, K. Tian, M. Pahlitzsch, J. Brenton, N. Ravindran, G. Butt, G. Malaguarnera, E.M. Normando, L. Guo, M.F. Cordeiro

**Affiliations:** a Department of Visual Neuroscience, UCL Institute of Ophthalmology, London EC1V 9EL, United Kingdom; b Western Eye Hospital, Imperial College London, United Kingdom

In issue 36 of Mitochondrion, an author was excluded from the article *‘Topical Coenzyme Q10 demonstrates mitochondrial-mediated neuroprotection in a rodent model of ocular hypertension’* on pages 114–123 at the time of publication. The missing author (D Stampoulis) is included below in the proper order and affiliation. The authors would like to apologise for any inconvenience caused.

Topical Coenzyme Q10 demonstrates mitochondrial-mediated neuroprotection in a rodent model of ocular hypertension

Davis BM^1^, Tian K^1^, Pahlitzsch M^1^, Brenton J^1^, Ravindran N^1^, Stampoulis D^4^, Butt G^1^, Malaguarnera G^1^, Normando EM^2^, Guo L^1^, Cordeiro MF^3^

^1^Department of Visual Neuroscience, UCL Institute of Ophthalmology, London EC1V 9EL, United Kingdom

^2^Department of Visual Neuroscience, UCL Institute of Ophthalmology, London EC1V 9EL, United Kingdom

^3^Department of Visual Neuroscience, UCL Institute of Ophthalmology, London EC1V 9EL, United Kingdom; Western Eye Hospital, Imperial College London, United Kingdom. Electronic address: m.cordeiro@ucl.ac.uk

^4^Department of Cell Biology, UCL Institute of Ophthalmology, London EC1V 9EL, United Kingdom

